# Accessible Transportation, Geographic Elevation, and Masticatory Ability Among Elderly Residents of a Rural Area

**DOI:** 10.3390/ijerph120707199

**Published:** 2015-06-26

**Authors:** Tsuyoshi Hamano, Kazumichi Tominaga, Miwako Takeda, Kristina Sundquist, Toru Nabika

**Affiliations:** 1 Center for Community-based Health Research and Education (COHRE), Organization for the Promotion of Project Research, Shimane University, 223-8 Enya-cho, Izumo, Shimane 693-8501, Japan; E-Mails: cohre1@med.shimane-u.ac.jp (M.T.); nabika@med.shimane-u.ac.jp (T.N.); 2 Tominaga Dental Office, 97-3 Yamada, Ohnan-cho, Ohchi, Shimane 696-0313, Japan; E-Mail: shika-t@ohtv.ne.jp; 3 Center for Primary Health Care Research, Lund University, Clinical Research Centre (CRC), Building 28, Floor 11, Jan Waldenströms Gata 35, Skåne University Hospital, SE-205 02 Malmö, Sweden; E-Mail: kristina.sundquist@med.lu.se; 4 Stanford Prevention Research Center, Stanford University, Medical School Office Building (MSOB), 251 Campus Drive MC 5411, Stanford, CA 94305, USA; 5 Department of Functional Pathology, Shimane University School of Medicine, 89-1 Enya-cho, Izumo, Shimane 693-8501, Japan

**Keywords:** masticatory ability, accessible transportation, elevation, mountainous area

## Abstract

Given that public transportation networks are often worse in rural areas than in urban areas, rural residents who do not drive can find it difficult to access health-promoting goods, services, and resources related to masticatory ability. Moreover, geographical location, assessed by elevation, could modify this association. The aim of this study was to test whether the association between access to transportation and masticatory ability varied by elevation. Data were collected from a cross-sectional study conducted in Mizuho and Iwami counties, Japan. Objective masticatory ability was evaluated using a test gummy jelly and elevation was estimated by the geographic information systems according to the participant’s address. After excluding subjects with missing data, 672 subjects (Mizuho = 401 and Iwami = 271) were analyzed. After adjustment for potential confounders, being a driver was not significantly associated with masticatory ability among elderly people living at low elevation (≤313 m) in Mizuho county. However, after the same adjustment, being a driver remained significantly associated with increased masticatory ability among elderly at high elevations. Similar findings were observed in Iwami county. Accessible transportation was significantly associated with increased mastication ability in elderly people living at high elevations, but not in those living at low elevations.

## 1. Introduction

Reduced masticatory disability in elderly people is a cause of reduced quality of life and increased cardiovascular mortality [1,2,3]. Several risk factors for masticatory ability have been postulated, including sociodemographic factors (e.g., age, gender, and income), lifestyle and health factors (e.g., physical activity, smoking, and current history of the disease), and oral health conditions (e.g., remaining teeth, use of dentures, and pattern of posterior occluding pairs of natural teeth) [4,5,6]. It is also important to note that the availability of accessible transportation could be considered as a potential risk factor for masticatory disability in rural areas [7].

Given that public transportation networks are often worse in rural areas than in urban areas, rural residents who do not drive can find it difficult to access health-promoting goods, services, and resources [7,8,9]. We therefore hypothesized that elderly people who do not drive have a lower masticatory ability than those who are drivers. Moreover, geographical location, as measured in terms of elevation, could modify the association between access to transportation, *i.e.*, drivers or not and masticatory ability. Hilly and mountainous areas occupy approximately 70% of Japan, and are defined as the regions extending from the outer plains to the mountains [10]. Health-promoting goods, services, and resources are substantially limited at higher elevations, as compared with their availability at lower elevations. To the best of our knowledge, previous studies have not investigated the hypothesized associations between masticatory ability, access to transportation, and elevation among elderly residents of rural areas. The aim of this study was to test whether the association between access to transportation and masticatory ability varied by elevation. Masticatory ability was evaluated using a test gummy jelly and access to transportation was assessed in terms of driving status.

## 2. Methods

### 2.1. Study Design

Data were collected from a cross-sectional study conducted in 2012. The present study was a part of the Shimane CoHRE Study, which was designed to examine the determinants of lifestyle-related diseases, including oral health [7,11]. The Shimane CoHRE study was conducted by Shimane University, Japan, in collaboration with a health examination program that involved in two counties, *i.e.*, Mizuho and Iwami, which include the town of Ohnan. These counties are located in a rural mountainous area in the southern part of Shimane prefecture, Japan.

A health examination program is available once a year for those between 40 and 74 years of age who are covered by National Health Insurance. The residents have two options when they wish to undergo a health examination program: they may participate in a group examination conducted at public health centers, or they may receive an individual examination at medical institutions. We were permitted to use the data on group examinations for our analysis. After excluding subjects with missing data (212 participants), we analyzed 401 participants in Mizuho county and 271 participants in Iwami county. The study protocol was approved by the ethics committee of Shimane University School of Medicine in 2010, and written informed consent was obtained from all participants.

### 2.2. Masticatory Ability

Objective masticatory ability was assessed using a gummy (a soft chewy candy). The subjects were instructed to chew a gummy jelly freely and after 15 s of chewing, the gummy jelly was collected immediately and the number of pieces was counted [12].

### 2.3. Elevation

Geographic information systems (ArcGIS software, version 10.0; Environmental Systems Research Institute, Redlands, CA, USA) was employed for database queries and used to estimate elevation based on the individual’s address. The elevation for each participant was assessed using the ArcGIS ready-to-use dataset of digital elevation models. Participants were divided into two groups on the basis of the median value of elevation in each county (median value in Mizuho county = 313 m and Iwami county = 212 m).

### 2.4. Other Measures

We considered the following variables in the analysis: age (years, analyzed as a continuous variable), gender (male *vs.* female), body mass index (BMI) calculated from self-reported weight and height (analyzed as a continuous variable), current smoking (yes *vs.* no), drinking alcohol (yes *vs.* no), physically active (engaged in physical activity regularly = yes *vs.* did not engage in physical activity regularly = no), use of medication for disease treatment (medication to reduce blood pressure, medication to reduce blood sugar or insulin injections, and medication to reduce cholesterol level), receiving a dental health check within the past year (yes *vs.* no), number of teeth (less than 20 *vs.* 20+), feeling stress or having an acute disease during the past 3 months (yes *vs.* no), having dentures (yes *vs.* no), having any oral health problems (yes *vs.* no), and accessible transportation (driver = yes *vs.* non-driver = no).

### 2.5. Statistical Analysis

Descriptive statistics were calculated. χ^2^ and *t*-tests were used to compare characteristics between the high- and low-elevation groups. Multivariable linear regression models were performed to derive regression coefficients (B), standard errors (SE), and *p*-values. Independent variables were coded as follows: gender (0 = male, 1 = female), number of teeth (0 = less than 19, 1 = 20 or more), and current smoking, drinking alcohol, regular physical activity, medication, use of dental health checks, feeling stress or having acute diseases, dentures, having any oral health problems, deriver (0 = no, 1 = yes). We run the same regression models for each county, *i.e.*, Mizuho and Iwami counties, to provide consistent results. *p*-values less than 0.05 were considered statistically significant. All statistical analyses were performed using IBM SPSS Statistics 20 (IBM Corporation, Tokyo, Japan).

## 3. Results

The characteristics of the study participants are shown in Table 1 and Table 2. In Mizuho county, there was no statistically significant difference between the low- and high-elevation groups in terms of masticatory ability (*i.e.*, the number of gummy jelly pieces) or accessible transportation (*i.e.*, driving status). Further, the low- and high-elevation groups did not differ significantly in terms of any of the following factors: age, gender, current smoking, drinking alcohol, regular physical activity, medication, BMI, use of dental health checks, number of teeth, stress or acute diseases, dentures, or oral health problems (Table 1). Similar patterns were observed in the participants of Iwami county (Table 2).

**Table 1:** Characteristics of the study participants in Mizuho county.

	Low Elevation (*n* = 136)		High Elevation (*n* = 135)		
	Number of Participants	% or Mean (SD)	Number of Participants	% or Mean (SD)	*p*-Value
Number of gummy jelly pieces	136	20.0 (17.5)	135	17.8 (14.0)	0.242
Driver, %	106	77.9	108	80.0	0.678
Age, years (SD)	136	66.5 (6.8)	135	65.3 (7.8)	0.180
Gender (male), %	62	45.6	53	39.3	0.292
Current smoking, %	16	11.8	13	9.6	0.570
Drinking alcohol, %	75	55.1	73	54.1	0.859
Regular physical activity, %	52	38.2	49	36.3	0.741
Medication					
To reduce blood pressure, %	50	36.8	47	34.8	0.738
To reduce blood sugar or insulin injections, %	11	8.1	5	3.7	0.126
To reduce cholesterol level, %	35	25.7	28	20.7	0.330
Body Mass Index, kg/m^2^	136	22.6 (2.9)	135	22.4 (3.1)	0.524
Use of dental health checks, %	74	54.4	78	57.8	0.577
Number of teeth (20 or more), %	54	39.7	49	36.3	0.563
Feeling stress or having acute disease, %	15	11.0	18	13.3	0.562
Dentures, %	67	49.3	58	43.0	0.298
Having any oral health problems, %	62	45.6	69	51.1	0.363

**Table 2:** Characteristics of the study participants in Iwami county.

	Low Elevation (*n* = 202)		High Elevation (*n* = 199)		
	Number of Participants	% or Mean (SD)	Number of Participants	% or Mean (SD)	*p-*Value
Number of gummy jelly pieces	202	19.4 (14.5)	199	22.1 (18.9)	0.119
Driver, %	158	78.2	159	79.9	0.679
Age, years (SD)	202	65.3 (8.1)	199	65.4 (8.1)	0.892
Gender (male), %	80	39.6	83	41.7	0.668
Current smoking, %	25	12.4	33	16.6	0.231
Drinking alcohol, %	102	50.5	111	55.8	0.289
Regular physical activity, %	56	27.7	48	24.1	0.411
Medication					
To reduce blood pressure, %	81	40.1	54	27.1	0.006
To reduce blood sugar or insulin injections, %	13	6.4	9	4.5	0.400
To reduce cholesterol level, %	41	20.3	39	19.6	0.861
Body Mass Index, kg/m^2^	202	22.7 (3.2)	199	23.1 (3.3)	0.273
Use of dental health checks, %	112	55.4	108	54.3	0.813
Number of teeth (20 or more), %	71	35.1	73	36.7	0.749
Feeling stress or having acute disease, %	30	14.9	31	15.6	0.840
Dentures, %	85	42.1	94	47.2	0.299
Having any oral health problems, %	115	56.9	114	57.3	0.943

Table 3 shows the results of the multivariable linear regression analysis in Mizuho county, as stratified by elevation. For the low-elevation group, being a driver was not significantly associated with masticatory ability (regression coefficient = 3.588, *p* = 0.257). Number of teeth and having dentures were significantly associated with masticatory ability (regression coefficient = 12.724, *p* ≤ 0.001 and regression coefficient = −12.818, *p* ≤ 0.001, respectively). For the high-elevation group, being a driver was significantly associated with increased masticatory ability (regression coefficient = 5.518, *p* = 0.032). Further, number of teeth, having dentures, and gender were significantly associated with masticatory ability (regression coefficient = 10.458, *p* ≤ 0.001; regression coefficient = −6.181, *p* = 0.019; and regression coefficient = −4.370, *p* = 0.038, respectively).

Table 4 shows the results of participants in Iwami county. Similar results were observed as shown in Mizuho county. For the low-elevation group, being a driver was not significantly associated with masticatory ability (regression coefficient = 4.040, *p* = 0.053). Number of teeth, gender, and current smoking were significantly associated with masticatory ability (regression coefficient = 15.805, *p* ≤ 0.001; regression coefficient = −5.036, *p* = 0.008; and regression coefficient = −6.495, *p* = 0.014, respectively). For the high-elevation group, being a driver was significantly associated with increased masticatory ability (regression coefficient = 5.909, *p* = 0.043). Also, number of teeth and use of dental health checks were significantly associated with masticatory ability (regression coefficient = 17.165, *p* ≤ 0.001 and regression coefficient = 5.046, *p* = 0.030, respectively).

**Table 3:** Multivariable linear regression analysis with masticatory ability as dependent variable in Mizuho county.

	Low Elevation (*n* = 136)				High Elevation (*n* = 135)			
	B	SE	t	*p*-Value	B	SE	t	*p*-Value
Age (years)	0.083	0.211	0.395	0.693	−0.219	0.150	−1.462	0.146
Gender (male *vs.* female)	−1.639	2.882	−0.569	0.571	−4.370	2.078	−2.103	0.038
Body mass index (kg/m^2^)	−0.303	0.431	−0.704	0.483	−0.425	0.309	−1.376	0.171
Current smoking (No *vs.* Yes)	−5.808	3.905	−1.488	0.139	−6.690	3.421	−1.956	0.053
Drinking alcohol (No *vs.* Yes)	1.991	2.497	0.797	0.427	−1.787	2.023	−0.884	0.379
Regular physical activity (No *vs.* Yes)	2.268	2.389	0.949	0.344	1.915	1.959	0.978	0.330
Medication (No *vs.* Yes)								
To reduce blood pressure	2.125	2.734	0.777	0.439	−1.364	2.446	−0.558	0.578
To reduce blood sugar or insulin injection	0.933	4.550	0.205	0.838	−5.772	4.999	−1.155	0.251
To reduce cholesterol level	−1.486	2.900	−0.513	0.609	−1.892	2.453	−0.771	0.442
Use of dental health checks (No *vs.* Yes)	4.886	2.328	2.099	0.038	2.365	2.033	1.164	0.247
Number of teeth (less than 19 *vs.* 20 or more)	12.724	3.486	3.650	<0.001	10.458	2.610	4.006	<0.001
Feeling stress or having acute disease (No *vs.* Yes)	−2.558	3.721	−0.688	0.493	0.385	2.834	0.136	0.892
Dentures (No *vs.* Yes)	−12.818	3.562	−3.599	<0.001	−6.181	2.590	−2.387	0.019
Having any oral health problems (No *vs.* Yes)	−3.427	2.424	−1.414	0.160	0.958	1.872	0.512	0.610
Driver (No *vs.* Yes)	3.588	3.148	1.140	0.257	5.518	2.542	2.171	0.032

**Table 4:** Multivariable linear regression analysis with masticatory ability as dependent variable in Iwami county.

	Low Elevation (*n* = 202)				High Elevation (*n* = 199)			
	B	SE	t	*p*-Value	B	SE	t	*p*-Value
Age (year)	−0.101	0.109	−0.925	0.356	−0.216	0.156	−1.382	0.196
Gender (male *vs.* female)	−5.036	1.867	−2.698	0.008	−2.565	2.764	−0.928	0.355
Body mass index (kg/m^2^)	−0.466	0.250	−1.860	0.064	−0.013	0.351	−0.036	0.971
Current smoking (No *vs.* Yes)	−6.495	2.624	−2.475	0.014	5.305	3.403	1.559	0.121
Drinking alcohol (No *vs.* Yes)	0.013	1.751	0.007	0.994	−2.514	2.567	−0.979	0.329
Regular physical activity (No *vs.* Yes)	2.131	1.796	1.186	0.237	−0.622	2.619	−0.237	0.813
Medication (No *vs.* Yes)								
To reduce blood pressure	−0.749	1.742	−0.430	0.668	−2.250	2.819	−0.798	0.426
To reduce blood sugar or insulin injection	−4.166	3.258	−1.279	0.203	0.139	5.591	0.025	0.980
To reduce cholesterol level	−2.209	2.103	−1.050	0.295	1.656	2.979	0.556	0.579
Use of dental health checks (No *vs.* Yes)	−0.844	1.599	−0.527	0.599	5.046	2.300	2.194	0.030
Number of teeth (less than 19 *vs.* 20 or more)	15.805	2.641	5.984	<0.001	17.165	3.012	5.699	<0.001
Feeling stress or having acute disease (No *vs.* Yes)	−0.405	2.253	−0.180	0.858	−2.880	3.165	−0.910	0.364
Dentures (No *vs.* Yes)	−2.566	2.562	−1.002	0.318	−5.930	3.012	−1.969	0.050
Having any oral health problems (No *vs.* Yes)	−0.819	1.602	−0.511	0.610	−0.647	2.292	−0.282	0.778
Driver (No *vs.* Yes)	4.040	2.072	1.950	0.053	5.909	2.906	2.034	0.043

## 4. Discussion

To the best of our knowledge, no study has examined the association between accessible transportation, *i.e.*, driving status, and masticatory ability according to the elevation. As expected, our results showed that accessible transportation was significantly associated with masticatory ability in elderly residents of high-elevation areas, but not elderly residents of low-elevation areas. This pattern of findings was evident in both counties. These results suggest that accessible transportation may be an important determinant of masticatory ability in elderly who live at relatively high elevations in rural areas. 

This study advances the recent debate regarding the potential risk factors for masticatory ability. For example, a previous study suggested that there are social factors that influence oral health [13]. A follow-up study conducted in Sweden found that the relative risk of reduced or impaired masticatory ability was higher in subject aged ≥65 years who lived alone, as compared with subjects of the same ages who lived with other persons [4]. These implications tend to support the different associations between accessible transportation and masticatory ability that we observed in the high- and low-elevation groups. The mechanism underlying this association is most likely of a complex nature, and causal inferences remain to be established. Considering that our subjects live in a rural mountainous area, non-driver subjects is more vulnerable to the effects of relatively limited access to health-promoting goods, services, and resources than drivers. Further studies are required to determine whether such health behaviors differ by accessible transportation in each elevation group.

Known determinants of masticatory ability include sociodemographic factors (e.g., age, gender, and income), lifestyle and health factors (e.g., physical activity, smoking, and current history of the disease), and oral health conditions. In addition, this study suggests that accessibility to the health-promoting goods, services, and resources could also determine masticatory ability. Health professionals should pay attention to local topography in order to formulate efficient health plans that can reduce masticatory disability and select appropriate target groups for interventions. For example, non-drivers who reside at higher elevations need education regarding healthy foods to help prevent masticatory disability. Moreover, promotion of oral health checkups would be considered a priority target for non-drivers living at higher elevations.

The present study has several strengths. To our knowledge, this is the first study to examine the association between accessible transportation and masticatory ability as stratified by elevation. In addition, our hypothesis was supported by results from both of the investigated counties. Further, masticatory ability was measured according to an objective method that has been validated more than subjective methods [12]. There are also a number of potential limitations to the current study. First, our data is not a representative sample: around 70% of participants had reached retirement age. A selection bias caused by non-respondents in each county was present and may have influenced the associations as shown in our results. Caution is therefore warranted in over-interpreting these findings; Second, misclassification may have occurred in the self-reported data as consequence of recall errors. However, we have no reason to believe that this potential bias differed between the residential areas; Third, our data did not allow for the assessment of other important factors for masticatory ability, such as familial composition, and occupation; Finally, the present study used a cross-sectional design that did not allow us to establish temporal ordering or causal relationships.

## 5. Conclusions

Our analysis indicated that accessible transportation was significantly associated with masticatory ability among persons living at higher elevations, but not among those living at lower elevations. These findings suggest that it is important to consider geographic location and accessibility to health-promoting goods, services, and resources when seeking to promote oral health in rural mountainous areas. Further longitudinal research is needed to confirm these findings and explore the underlying mechanisms.
